# Correction: Estimating the proportion of clinically suspected cholera cases that are true Vibrio cholerae infections: A systematic review and meta-analysis

**DOI:** 10.1371/journal.pmed.1004743

**Published:** 2025-09-11

**Authors:** Kirsten E. Wiens, Hanmeng Xu, Kaiyue Zou, John Mwaba, Justin Lessler, Espoir Bwenge Malembaka, Maya N. Demby, Godfrey Bwire, Firdausi Qadri, Elizabeth C. Lee, Andrew S. Azman

There are errors in the Funding statement. The correct Funding statement is as follows: This work was supported by the Bill and Melinda Gates Foundation (https://www.gatesfoundation.org/) [grant number OPP1171700 to A.S.A.] and the National Institute of Allergy and Infectious Disease (https://www.niaid.nih.gov/) [grant number AI135115-01A1 to A.S.A. and K22AI168389 to K.E.W.]. The funders had no role in study design, data collection and analysis, decision to publish, or preparation of the manuscript.
