# Supplementary material for: Estimating the proportion of clinically suspected cholera cases that are true Vibrio cholerae infections: A systematic review and meta-analysis
Source: PLoS Med. 2023 Sep 14;20(9):e1004286. doi: 10.1371/journal.pmed.1004286 (PMC10538743; doi:10.1371/journal.pmed.1004286)
Supplement: Attachment — Submitted filename: Cholera_positivity_RTR.docx [file pmed.1004286.s004.docx]

**Responses to Reviewers and Editors**

Dear Dr. Azman,

Thank you very much for submitting your manuscript "Towards estimating true cholera burden: a systematic review and meta-analysis of Vibrio cholerae positivity" (PMEDICINE-D-22-03502R1) for consideration at PLOS Medicine.

Your paper was evaluated by a senior editor and discussed among all the editors here. It was also discussed with an academic editor with relevant expertise, and sent to independent reviewers, including a statistical reviewer. The reviews are appended at the bottom of this email and any accompanying reviewer attachments can be seen via the link below:

In light of these reviews, I am afraid that we will not be able to accept the manuscript for publication in the journal in its current form, but we would like to consider a revised version that addresses the reviewers' and editors' comments. Obviously we cannot make any decision about publication until we have seen the revised manuscript and your response, and we plan to seek re-review by one or more of the reviewers.

In revising the manuscript for further consideration, your revisions should address the specific points made by each reviewer and the editors. Please also check the guidelines for revised papers at [http://journals.plos.org/plosmedicine/s/revising-your-manuscript](https://nam10.safelinks.protection.outlook.com/?url=http%3A%2F%2Fjournals.plos.org%2Fplosmedicine%2Fs%2Frevising-your-manuscript&data=05%7C01%7Ckirsten.wiens%40temple.edu%7Cd4d961189fb0451bcfff08db3f49debb%7C716e81efb52244738e3110bd02ccf6e5%7C0%7C0%7C638173358737084023%7CUnknown%7CTWFpbGZsb3d8eyJWIjoiMC4wLjAwMDAiLCJQIjoiV2luMzIiLCJBTiI6Ik1haWwiLCJXVCI6Mn0%3D%7C3000%7C%7C%7C&sdata=PtraCccpWJdKOPWp77u4aMmYTmYZEAETwx%2B50Fv63sI%3D&reserved=0) for any that apply to your paper. In your rebuttal letter you should indicate your response to the reviewers' and editors' comments, the changes you have made in the manuscript, and include either an excerpt of the revised text or the location (eg: page and line number) where each change can be found. Please submit a clean version of the paper as the main article file; a version with changes marked should be uploaded as a marked up manuscript.

**Response**: We have checked the guidelines for revised papers.

In addition, we request that you upload any figures associated with your paper as individual TIF or EPS files with 300dpi resolution at resubmission; please read our figure guidelines for more information on our requirements: [http://journals.plos.org/plosmedicine/s/figures](https://nam10.safelinks.protection.outlook.com/?url=http%3A%2F%2Fjournals.plos.org%2Fplosmedicine%2Fs%2Ffigures&data=05%7C01%7Ckirsten.wiens%40temple.edu%7Cd4d961189fb0451bcfff08db3f49debb%7C716e81efb52244738e3110bd02ccf6e5%7C0%7C0%7C638173358737084023%7CUnknown%7CTWFpbGZsb3d8eyJWIjoiMC4wLjAwMDAiLCJQIjoiV2luMzIiLCJBTiI6Ik1haWwiLCJXVCI6Mn0%3D%7C3000%7C%7C%7C&sdata=6CTLCUlfQK1jIsoNVScW3pmAltho9TQSWzoIQkGXwuk%3D&reserved=0). While revising your submission, please upload your figure files to the PACE digital diagnostic tool, [https://pacev2.apexcovantage.com/](https://nam10.safelinks.protection.outlook.com/?url=https%3A%2F%2Fpacev2.apexcovantage.com%2F&data=05%7C01%7Ckirsten.wiens%40temple.edu%7Cd4d961189fb0451bcfff08db3f49debb%7C716e81efb52244738e3110bd02ccf6e5%7C0%7C0%7C638173358737084023%7CUnknown%7CTWFpbGZsb3d8eyJWIjoiMC4wLjAwMDAiLCJQIjoiV2luMzIiLCJBTiI6Ik1haWwiLCJXVCI6Mn0%3D%7C3000%7C%7C%7C&sdata=eJNKSenzXKgR%2FpDAgMcjqxLGTljfXY8RinMUofRSkiE%3D&reserved=0). PACE helps ensure that figures meet PLOS requirements. To use PACE, you must first register as a user. Then, login and navigate to the UPLOAD tab, where you will find detailed instructions on how to use the tool. If you encounter any issues or have any questions when using PACE, please email us at [PLOSMedicine@plos.org](mailto:PLOSMedicine@plos.org).

**Response**: We have checked the figures using the PACE tool. The figures uploaded with this revision are tiff files that have been PACE-corrected, and we checked them all for clarity.

We expect to receive your revised manuscript by May 08 2023 11:59PM. Please email us (plosmedicine@plos.org) if you have any questions or concerns.

***Please note while forming your response, if your article is accepted, you may have the opportunity to make the peer review history publicly available. The record will include editor decision letters (with reviews) and your responses to reviewer comments. If eligible, we will contact you to opt in or out.***

We ask every co-author listed on the manuscript to fill in a contributing author statement, making sure to declare all competing interests. If any of the co-authors have not filled in the statement, we will remind them to do so when the paper is revised. If all statements are not completed in a timely fashion this could hold up the re-review process. If new competing interests are declared later in the revision process, this may also hold up the submission. Should there be a problem getting one of your co-authors to fill in a statement we will be in contact. YOU MUST NOT ADD OR REMOVE AUTHORS UNLESS YOU HAVE ALERTED THE EDITOR HANDLING THE MANUSCRIPT TO THE CHANGE AND THEY SPECIFICALLY HAVE AGREED TO IT. You can see our competing interests policy here: [http://journals.plos.org/plosmedicine/s/competing-interests](https://nam10.safelinks.protection.outlook.com/?url=http%3A%2F%2Fjournals.plos.org%2Fplosmedicine%2Fs%2Fcompeting-interests&data=05%7C01%7Ckirsten.wiens%40temple.edu%7Cd4d961189fb0451bcfff08db3f49debb%7C716e81efb52244738e3110bd02ccf6e5%7C0%7C0%7C638173358737084023%7CUnknown%7CTWFpbGZsb3d8eyJWIjoiMC4wLjAwMDAiLCJQIjoiV2luMzIiLCJBTiI6Ik1haWwiLCJXVCI6Mn0%3D%7C3000%7C%7C%7C&sdata=HDV7m1g1PEKrAk6OEPuoQMzaQd2GF680kWQtiOEhFVs%3D&reserved=0).

**Response**: Thank you for the reminder. We have not changed the author list and none of the authors have declared competing interests. If any of the authors have not yet filled in their statements, please let us know and we can send them an additional email.

Please use the following link to submit the revised manuscript:

[https://www.editorialmanager.com/pmedicine/](https://nam10.safelinks.protection.outlook.com/?url=https%3A%2F%2Fwww.editorialmanager.com%2Fpmedicine%2F&data=05%7C01%7Ckirsten.wiens%40temple.edu%7Cd4d961189fb0451bcfff08db3f49debb%7C716e81efb52244738e3110bd02ccf6e5%7C0%7C0%7C638173358737084023%7CUnknown%7CTWFpbGZsb3d8eyJWIjoiMC4wLjAwMDAiLCJQIjoiV2luMzIiLCJBTiI6Ik1haWwiLCJXVCI6Mn0%3D%7C3000%7C%7C%7C&sdata=ixFxILhvK6LSctrK7tW89c3BplmrCuRbQjRTx%2F19vmE%3D&reserved=0)

Your article can be found in the "Submissions Needing Revision" folder.

To enhance the reproducibility of your results, we recommend that you deposit your laboratory protocols in protocols.io, where a protocol can be assigned its own identifier (DOI) such that it can be cited independently in the future. Additionally, PLOS ONE offers an option to publish peer-reviewed clinical study protocols. Read more information on sharing protocols at [https://plos.org/protocols?utm_medium=editorial-email&utm_source=authorletters&utm_campaign=protocols](https://nam10.safelinks.protection.outlook.com/?url=https%3A%2F%2Fplos.org%2Fprotocols%3Futm_medium%3Deditorial-email%26utm_source%3Dauthorletters%26utm_campaign%3Dprotocols&data=05%7C01%7Ckirsten.wiens%40temple.edu%7Cd4d961189fb0451bcfff08db3f49debb%7C716e81efb52244738e3110bd02ccf6e5%7C0%7C0%7C638173358737084023%7CUnknown%7CTWFpbGZsb3d8eyJWIjoiMC4wLjAwMDAiLCJQIjoiV2luMzIiLCJBTiI6Ik1haWwiLCJXVCI6Mn0%3D%7C3000%7C%7C%7C&sdata=IyNAg6wWwlrS8c%2BAW5yAUCTNZX5JNYWbJO2fF8VIqsg%3D&reserved=0)

Please ensure that the paper adheres to the PLOS Data Availability Policy (see [http://journals.plos.org/plosmedicine/s/data-availability)](https://nam10.safelinks.protection.outlook.com/?url=http%3A%2F%2Fjournals.plos.org%2Fplosmedicine%2Fs%2Fdata-availability)&data=05%7C01%7Ckirsten.wiens%40temple.edu%7Cd4d961189fb0451bcfff08db3f49debb%7C716e81efb52244738e3110bd02ccf6e5%7C0%7C0%7C638173358737084023%7CUnknown%7CTWFpbGZsb3d8eyJWIjoiMC4wLjAwMDAiLCJQIjoiV2luMzIiLCJBTiI6Ik1haWwiLCJXVCI6Mn0%3D%7C3000%7C%7C%7C&sdata=saMJ1HOgKSkgVKrOiDz1KZcay%2BMy7OH4UUAN%2FD0najo%3D&reserved=0), which requires that all data underlying the study's findings be provided in a repository or as Supporting Information. For data residing with a third party, authors are required to provide instructions with contact information for obtaining the data. PLOS journals do not allow statements supported by "data not shown" or "unpublished results." For such statements, authors must provide supporting data or cite public sources that include it.

**Response**: All the data collected and generated in this study is publicly available within the manuscript and in the linked GitHub repository.

We look forward to receiving your revised manuscript.

Sincerely,

Philippa Dodd, MBBS MRCP PhD

PLOS Medicine

plosmedicine.org

-----------------------------------------------------------

Requests from the editors:

GENERAL

Please respond to all editor and reviewer comments detailed below in full.

* Please report your SR/MA according to the PRISMA guidelines provided at the EQUATOR site.

[http://www.equator-network.org/reporting-guidelines/prisma/](https://nam10.safelinks.protection.outlook.com/?url=http%3A%2F%2Fwww.equator-network.org%2Freporting-guidelines%2Fprisma%2F&data=05%7C01%7Ckirsten.wiens%40temple.edu%7Cd4d961189fb0451bcfff08db3f49debb%7C716e81efb52244738e3110bd02ccf6e5%7C0%7C0%7C638173358737084023%7CUnknown%7CTWFpbGZsb3d8eyJWIjoiMC4wLjAwMDAiLCJQIjoiV2luMzIiLCJBTiI6Ik1haWwiLCJXVCI6Mn0%3D%7C3000%7C%7C%7C&sdata=5%2BAGd5CvrDG4C5sZl1S%2BzyJfloLVRVAz6pUSS%2FFcVHg%3D&reserved=0)

- Please provide the completed PRISMA checklist. When completing the checklist, please use section and paragraph numbers, rather than page or line numbers, as these often change in the event of publication.

- Please add the following statement, or similar, to the Methods: "This study is reported as per the Preferred Reporting Items for Systematic Reviews and Meta-Analyses (PRISMA) guideline (S1 Checklist)."

**Response**: We have included the PRISMA checklist as a supplemental document in this revision and added the PRISMA statement and additional information as per PRISMA guidelines in the final paragraph of the methods “Systematic review” section.

** Was the study registered with PROSPERO? Please provide details.

**Response**: No, the study was not registered with PROSPERO, and we are not able to register in now because extraction has already been completed. We have indicated this in the final paragraph of the methods “Systematic review” section.

*** Please update your search to the present time – PLOS Medicine requires that all systematic reviews are updated to within 6 months of an anticipated publication date.

**Response**: We updated the search on April 19, 2023. We have included the details of the updated in the search in the methods “Systematic review” section first paragraph and in the Supplementary Methods on page 2. In brief, this resulted in an additional 787 studies to screen, of which 7 were included with data extracted.

COMMENTS FROM THE ACADEMIC EDITOR

Congrats on collating and synthesizing these important data. Would your team be able to elaborate further on the utility of using suspect cholera case definitions in outbreak and non-outbreak settings? It seems that these case definitions have performed well during outbreaks (with some recommendations made in lines 258-270) but that laboratory confirmation may be needed for sporadic cases?

**Response**: Thank you for these comments. We have expanded on the utility of suspected cholera case definitions and laboratory confirmation in the discussion paragraph four, which focuses on policy implications.

COMMENTS FROM THE EDITORS

ABSTRACT

Please report your abstract according to PRISMA for abstracts, following the PLOS Medicine abstract structure (Background, Methods and Findings, Conclusions) [http://www.plosmedicine.org/article/info:doi/10.1371/journal.pmed.1001419](https://nam10.safelinks.protection.outlook.com/?url=http%3A%2F%2Fwww.plosmedicine.org%2Farticle%2Finfo%3Adoi%2F10.1371%2Fjournal.pmed.1001419&data=05%7C01%7Ckirsten.wiens%40temple.edu%7Cd4d961189fb0451bcfff08db3f49debb%7C716e81efb52244738e3110bd02ccf6e5%7C0%7C0%7C638173358737084023%7CUnknown%7CTWFpbGZsb3d8eyJWIjoiMC4wLjAwMDAiLCJQIjoiV2luMzIiLCJBTiI6Ik1haWwiLCJXVCI6Mn0%3D%7C3000%7C%7C%7C&sdata=Z0vFDx2pJhL6uum%2BaNGYsXEbrvPsg1rsj50MMyFOxLc%3D&reserved=0)

**Response**: We have updated our abstract to ensure compliance with both PRISMA and PLOS Medicine guidelines.

Please provide the specific start and end dates of your search, data sources, types of study designs included, eligibility criteria, and synthesis/appraisal methods.

**Response**: Some of these details were already included in the methods, and we have now expanded on them in the methods “Systematic review” section.

Please ensure that all numbers presented in the abstract are present and identical to numbers presented in the main manuscript text.

**Response**: We have double checked that the numbers match.

Please quantify the main results p values as well as with 95% CIs. Please report as p <0.001 or where higher as p=0.002, for example. Suggest separating upper and lower bounds of 95% CIs with commas instead of hyphens as these can be confused with negative values. For example, lines 56-57, suggest “…1.64 (95% Credible Interval: 1.06,2.52; p< or =)

**Response**: We have added p values for all Spearman correlation analyses in the results where we report confidence intervals.

The main results were estimated using a Bayesian framework and we did not calculate p values that correspond to the 95% Credible Intervals. For the odds ratios, Credible Intervals that do not cross 1 are considered significant. For overall estimates of the proportion positive, “significance” statements are not appropriate because we are not making comparisons or doing any formal hypothesis testing. We have included a brief description of this in the methods paragraph “*Estimating* V. cholerae *positivity and sources of heterogeneity”* section first paragraph. We also updated the CrIs to be separated by commas.

Please include numerators and denominators used to derive percentages

**Response**: We have added numerators and denominators throughout.

Line 56 – do you present odds ratios here? Please clarify/define numerical values for the reader

**Response**: Yes, we have clarified this in the abstract results.

Please include any important variables that are adjusted for in the analyses.

**Response**: We have added this.

In the last sentence of the Abstract Methods and Findings section, please describe the main limitation(s) of the study's methodology.

**Response**: We have included the main limitations in the abstract.

Abstract Conclusions:

Please address the study implications without overreaching what can be concluded from the data; the phrase "In this study, we observed ..." may be useful.

Please interpret the study based on the results presented in the abstract, emphasizing what is new without overstating your conclusions.

Please avoid vague statements such as "these results have major implications for policy/clinical care". Mention only specific implications substantiated by the results.

**Response**: We have rephrased the abstract conclusions to include the phrase “In this study, we found that…”.

AUTHOR SUMMARY

At this stage, we ask that you include a short, non-technical Author Summary of your research to make findings accessible to a wide audience that includes both scientists and non-scientists. The author summary should consist of 2-3 succinct bullet points under each of the following headings:

• Why Was This Study Done? Authors should reflect on what was known about the topic before the research was published and why the research was needed.

• What Did the Researchers Do and Find? Authors should briefly describe the study design that was used and the study’s major findings. Do include the headline numbers from the study, such as the sample size and key findings.

• What Do These Findings Mean? Authors should reflect on the new knowledge generated by the research and the implications for practice, research, policy, or public health. Authors should also consider how the interpretation of the study’s findings may be affected by the study limitations.

The Author Summary should immediately follow the Abstract in your revised manuscript. This text is subject to editorial change and should be distinct from the scientific abstract. Please see our author guidelines for more information: [https://journals.plos.org/plosmedicine/s/revising-your-manuscript#loc-author-summary](https://nam10.safelinks.protection.outlook.com/?url=https%3A%2F%2Fjournals.plos.org%2Fplosmedicine%2Fs%2Frevising-your-manuscript%23loc-author-summary&data=05%7C01%7Ckirsten.wiens%40temple.edu%7Cd4d961189fb0451bcfff08db3f49debb%7C716e81efb52244738e3110bd02ccf6e5%7C0%7C0%7C638173358737084023%7CUnknown%7CTWFpbGZsb3d8eyJWIjoiMC4wLjAwMDAiLCJQIjoiV2luMzIiLCJBTiI6Ik1haWwiLCJXVCI6Mn0%3D%7C3000%7C%7C%7C&sdata=uIUavyjlc2fYJVB4B5kPQiefwGYzfCTa12R7qKCP3d0%3D&reserved=0)

**Response**: We have included an author summary following the above guidelines.

INTRODUCTION

The editorial team agree that it would be helpful to justify/clarify how measuring the ‘burden’ of cholera is helpful considering it occurs most often in the context of sporadic outbreaks (peaks & troughs).

**Response**: Thank you for this comment. Cholera persists in both endemic regions with persistent new cases as well as epidemic regions with sporadic outbreaks. An average ‘burden’ of cholera across multiple years allows us to identify ‘hotspots’, especially in endemic areas. The Global Task Force for Cholera Control currently defines these as areas of high incidence, persistence, mortality, and/or cholera test positivity (reference: <https://www.gtfcc.org/resources/identification-of-priority-areas-for-multisectoral-interventions-pamis-for-cholera-control/>). These ‘hotspots’ are helpful primarily because they are one of the best metrics available to inform global distribution of limited vaccine supply. We have expanded further on this in the final paragraph of the introduction.

Please indicate whether your study is novel and how you determined that. If there has been a previous systematic review of the evidence, please refer to and reference that review and indicate whether it supports the need for your study.

**Response**: In the first paragraph of the introduction, we described some of the previous individual studies that sought to address the gaps in our understanding of suspected cholera positivity rates, with varying results. To our knowledge, this is the first systematic review of cholera positivity and associated factors. We determined this through literature review and discussion with experts. We have now described this in the introduction second paragraph.

METHODS and RESULTS

Please justify why your search start date was 2000

**Response**: We restricted our search to studies conducted since 2000 to make sure that our estimates reflect relatively recent patterns in cholera positivity. We have expanded on this in the methods systematic review section first paragraph.

As above please ensure you update the search to the present time.

**Response**: We updated the search on April 19, 2023. These details are included in methods paragraph “Systematic review” section and Supplementary Methods and all figures and results have been updated with the additional eight included studies.

PLOS Medicine encourages inclusion of all non-English language studies. Please justify why studies reported in languages other than “...English, French, Spanish, and Chinese…” were excluded (line 117).

**Response**: We restricted the languages to those that our team was comfortable reviewing with some proficiency. Given the nuances of some of our exclusion criteria, we did not feel comfortable relying on services like Google Translate for languages such as Arabic which our team had no experience with. This resulted in exclusion of a single study, written in Arabic (Figure 1), and so this has very likely not impacted our overall results or conclusions. We added this in the methods systematic review section first paragraph.

Studies reported on Medrix are not peer reviewed publications. It is not clear how many (if any) were included in the analyses. We suggest that these are excluded from the main analyses.

Please be reminded to update the manuscript accordingly including the PRISMA flowchart, figures, tables as necessary

**Response**: Only one study from the original analysis was a non-peer reviewed preprint (https://www.medrxiv.org/content/10.1101/2021.08.20.21262313v1). A similar paper has now been published (https://journals.plos.org/plosntds/article?id=10.1371/journal.pntd.0009046), but no longer meets our inclusion criteria because it does not provide cholera positivity data. Therefore, we have removed this study from the analyses and updated the manuscript.

In addition, we have added notes in the methods “Systematic review” section first paragraph and Supplementary Methods on page 2 indicating that, even though our searches and screenings included pre-prints, we excluded all studies that had not been peer reviewed by the time of the updated search.

As above, please include p values where you report 95% CIs. Please provide the statistical tests used to determine p values. Please report as p <0.001 or where higher as p=0.002, for example.

Please ensure consistency when reporting upper and lower 95% CI bounds in the abstract hyhens are used, here the word “to” when negative values are reported. We suggest the use of commas throughout.

**Response**: We have added p values in the format requested for all Spearman correlation analyses in the results where we report 95% Confidence Intervals.

As mentioned above, the main results were estimated using a Bayesian framework and so we do not have p values that correspond to the 95% Credible Intervals.

When referring to ‘odds’ do you mean odds ratio (OR)? Please clarify and define numerical values as necessary for the reader.

**Response**: Yes, we have clarified this in the methods “Data analysis” “*Estimating* V. cholerae *positivity and sources of heterogeneity”* section first paragraph.

FIGURES

To make your figures more accessible to those with colour blindness, please consider avoiding the use of green and/or red.

**Response**: We have used color-blind-friendly palettes throughout to the best of our knowledge. For example, we used teal and orange instead of green and red based on the colour-blind friend palette “dark2” available from RColorBrewer Package. Please let us know if there are specific combinations that we should change in any of the figures.

Figure 1 – details here are inconsistent with those in the abstract and main manuscript text, please revise in line with the above comments.

**Response**: We have corrected this, and all numbers are revised with the inclusion of additional studies in the updated literature search.

Figure 2 - Please clearly indicate in the figure caption the meaning of the boxes and whiskers

**Response**: We have included this in the figure captions.

Figure 3 – Please clearly indicate in the figure caption the meaning of the dots and lines

**Response**: We have included this in the figure captions.

TABLES

Table 1 – please define PCR, RDT, please define the total number of studies as n= in the column header. Table 1 caption – in general this is a bit confusing “study-country-periods” is mentioned more than once but not in the table, what does it mean? Please revise/clarify for the reader such that the table contents are clearly defined without the need to refer to the manuscript text

There is 1 study defined as ‘other’ considering it is a single study would it be helpful to simply define it?

**Response**: We have made these revisions in Table 1.

DISCUSSION

Please present and organize the Discussion as follows: a short, clear summary of the article's findings; what the study adds to existing research and where and why the results may differ from previous research; strengths and limitations of the study; implications and next steps for research, clinical practice, and/or public policy; one-paragraph conclusion. Please avoid the use of sub-headings such that discussion reads as a single continuous piece of prose.

**Response**: We have moved the “policy” paragraph to after the strengths and limitations paragraph and expanded on what the study adds to existing research after the initial short summary of the findings.

REFERENCES

In the bibliography, please ensure that up to, but no more than, 6 author names are listed followed by et al, in the event that more that 6 authors contribute to an individual study. Journal name abbreviations should be those found in the National Center for Biotechnology Information (NCBI) databases.

Please see our website for other reference guidelines [https://journals.plos.org/plosmedicine/s/submission-guidelines#loc-references](https://nam10.safelinks.protection.outlook.com/?url=https%3A%2F%2Fjournals.plos.org%2Fplosmedicine%2Fs%2Fsubmission-guidelines%23loc-references&data=05%7C01%7Ckirsten.wiens%40temple.edu%7Cd4d961189fb0451bcfff08db3f49debb%7C716e81efb52244738e3110bd02ccf6e5%7C0%7C0%7C638173358737084023%7CUnknown%7CTWFpbGZsb3d8eyJWIjoiMC4wLjAwMDAiLCJQIjoiV2luMzIiLCJBTiI6Ik1haWwiLCJXVCI6Mn0%3D%7C3000%7C%7C%7C&sdata=7p07SWaqyIQttnNFWFnBtU05O%2BGzJyeTNq9wSJ8mQWw%3D&reserved=0)

**Response**: We have double checked that the references conform to the above guidelines.

SUPPLEMENTARY FILES

SUPP. METHODS

please ensure consistency with main manuscript text and figures

**Response**: We have double checked that the text and figures are consistent and have updated all with the new search results.

SUPP. FIGURES

Figure 1 & 2 – Please confirm that the appropriate usage rights apply to the use of this map. Please see our guidelines for map images: [https://journals.plos.org/plosmedicine/s/figures#loc-maps](https://nam10.safelinks.protection.outlook.com/?url=https%3A%2F%2Fjournals.plos.org%2Fplosmedicine%2Fs%2Ffigures%23loc-maps&data=05%7C01%7Ckirsten.wiens%40temple.edu%7Cd4d961189fb0451bcfff08db3f49debb%7C716e81efb52244738e3110bd02ccf6e5%7C0%7C0%7C638173358737084023%7CUnknown%7CTWFpbGZsb3d8eyJWIjoiMC4wLjAwMDAiLCJQIjoiV2luMzIiLCJBTiI6Ik1haWwiLCJXVCI6Mn0%3D%7C3000%7C%7C%7C&sdata=Ew%2F9VHxYRmv8fU%2BAkN0kJmV3uWTrV4dsRNL2Ti641OY%3D&reserved=0)

**Response**: We have double checked that the appropriate usage rights apply. These maps were created by our study team and the GADM shapefiles are available openly through creative commons: <https://gadm.org/data.html>. The Natural Earth shapefiles we used are also in the public domain and freely available to use: <https://www.naturalearthdata.com/about/terms-of-use/>

SUPP. TABLES

Please ensure all abbreviations are defined in the captions or an appropriate footnote (PCR/RDT, for example)

Please consider the use of commas to separate upper and lower bounds as hyphens can be confused with reporting of negative values.

**Response**: We have formatted as requested.

Comments from the reviewers:

Reviewer #1: Thanks for the opportunity to review your manuscript. My role is as a statistical reviewer, so my review concentrates on the study design, data, and analysis that are presented. I have put general questions first, followed by queries relevant to a specific section of the manuscript (with a page/line reference).

This study is a systematic review and meta-analysis that examines test positivity for cholera. The systematic review is broad and includes a large pool of studies that met quality requirements. A Bayesian approach to test positivity is used, and includes an assessment of factors (surveillance, test type etc.) that may be associated with test positivity. This is out of my own usual working area, but I was convinced that an understanding of what affects test positivity rates is necessary background to make improvements to the quality of cholera testing. There were many overlapping studies (where the same patients contribute the same data to different studies), and this was dealt with by selecting the more desirable study (in terms of quality and size). While not perfect, this seems to be a reasonable way of dealing with the overlap. The analysis is Bayesian latent class model that takes into account the correlation of measures of test performance within the same study. The manuscript is clearly written, and I found the supplementary materials were excellent in helping me to understand the analysis (nice figures e.g. S4).

**Response**: We thank the reviewer for these thoughtful comments.

The methods sections mentions has JAGs being used for analysis, but in the supplementary methods Stan is also mentioned. To clarify, was JAGS used for the latent class part of the analysis and Stan for the hierarchical meta-analysis? This is what it looks like from the github site.

**Response**: Yes, thank you for catching this. We have clarified in the methods section “*Estimating* V. cholerae *positivity and sources of heterogeneity*” first paragraph that Stan was used for the hierarchical meta-analysis and included the appropriate citations.

Are there terms to describe the types of cholera studies that met the inclusion criteria? E.g 'high quality' or 'population-based'?

**Response**: Thank you for this question. We did not include or exclude studies based on study type. We included both “high quality” or “population-based” as well as “low quality” or “convenience samples”, if they tested at least 10 suspected cholera cases. We clarified this in the last sentence of the Systematic Review section in the methods. Our rationale for this was that in most outbreak settings high quality sampling is not feasible. We wanted the opportunity to specifically test for differences in positivity by setting and sampling approach.

The analysis uses beta(1,1,) priors, i.e. 'flat prior', where all values of prevalence, sensitivity, and specificity are equally likely with varying upper and lower bounds. I would argue that this is probably not strictly 'uninformative' and it might be better to describe them differently, e.g. flat prior.

**Response**: We have rephrased our methods to describe these as “flat” priors as suggested.

I can follow the truncation bounds for some of the priors, but not others. For example, I wasn't clear why the lower bound of truncation for specificity was different between PCR and RDT. Some explanation of why these truncation points were chosen for each parameter would be helpful.

**Response**: We have also included further justification for the truncation on the priors in the methods section “Data analysis” second paragraph.

R-hat is checked for model convergence, where any other diagnostic checks from analysis (e.g. trace plots, divergence plots) performed?

**Response**: Yes, we checked R-hat and traceplots for convergence. We have revised the methods “Data analysis” section to include this.

In updating the analyses with new data that was published through April 2023, we found that a couple of our models did not converge perfectly. We ran several different tests, including increasing sampling iterations and reparametrizing the model, but were not able to get all parameters to fully converge, especially the model that did not include any covariates. We believe this is likely due to the large heterogeneity in the data.

We discussed our modeling approach and the diagnostics with a Stan expert at JHU (Dr. Javier Perez-Saez, included in the acknowledgments). Based on his recommendations, we decided to remove the component where we pool adjusted positivity across all the studies (which was essentially double pooling) to get a mean underlying positivity. Previously we were using the inferred mean of this pooling distribution to get the percent positive, and now that we removed this distribution we focus, perhaps more conventionally, on the global intercept as the target of inference. We also integrate over the study-level random effects in the updated manuscript to provide appropriate marginal estimates of positivity. We have included details in Supplementary Methods page 3 section "Statistical model”.

In addition, we modified our approach for incorporating test performance uncertainty by taking a random draw of sensitivity and specificity in each iteration of the Stan model, rather than looping over 1000 draws in each iteration. This greatly improved the computational efficiency of the models and substantially reduced the number of required iterations. With these adjustments, our models converged for all parameters.

Finally, in our revision we used the model adjusted only for diagnostic test sensitivity and specificity to estimate *V. cholerae* positivity across all studies. We considered reporting *V. cholerae* positivity for models additionally adjusted for study methodology (as in our previous submission), post-stratified using the proportion of studies in the literature review that fell into each stratum, but we did not feel that this estimate had a meaningful interpretation. Instead, we report *V. cholerae* positivity estimates by different covariate strata. These results are shown in Table S4 and Figure 3, and methods are detailed in the Supplementary Methods section “Estimation of *V. cholerae* positivity overall and by study methodology”.

Ultimately, this improved approach with the new data does not change our conclusions, but it does increase the uncertainty around our estimates. This makes sense considering the heterogeneity in the data, and we have emphasized this variation more strongly in the revision.

Table S1 - should the last row 'Specificity of PCR uspec(RD)' actually be 'Specificity of RDT'?

**Response**: Yes, thank you for catching this. We have corrected it.

Figure 2. With the number of studies contributing data I found it hard to get an understanding about differences in distribution of positivity according to the stratifying variables and quality of sampling. I wonder if something similar but with probability density plots (or violin or ridgeline plots) instead of box plots might allow more information to come out of this visualisation.

**Response**: Thank you for this comment. We agree that the figure was overly complicated, with the differences by quality of sampling difficult to discern in panels A,C, and D. We explored using alternatives to the box plots and have provided an example with violin plots below. We still found this difficult to interpret and so in the revision we have simplified Figure 2 to only show mean and points by the stratifying variables and not additionally by sampling quality. This is also consistent with how we describe these results in the manuscript text.

Reviewer #2:

General remark:

The study covers a relevant topic and is well written in most parts. However, the manuscript has some major limitations. This systematic review and meta-analysis does not fulfil the requirements of the Preferred Reporting Items for Systematic Reviews and Meta-Analyses guidelines that may help to improve the manuscript. Please add a prisma cheklist as supplemantary file.

**Response**: Thank you for this comment. We have included a PRISMA checklist as a supplementary file, and have included additional details in the abstract, methods, results, and discussion in compliance with PRISMA guidelines.

Reviewer #3: Thank you for considering me as a reviewer for this very interesting and timely analysis. Given the surge of cholera globally in the recent years, the study is contextual and adds to the evidence base. The key finding, of significantly higher odds of detecting cholera during outbreak-linked surveillance is intuitively expected, but pegging an estimated value will possibly help in understanding reported cases better (especially in outbreak-prone areas).

**Response**: Thank you for these kind comments.

The discussion section lays out the shortcomings in the analysis, but it would be interesting if the authors could reflect on the policy implications of their findings, specifically, how can these findings be used by program managers and policymakers in cholera endemic and outbreak prone countries. This is especially important considering the rapid surge and spread of cholera in countries which have not seen a significant burden of cholera cases for several years (some even for decades).

**Response**: In response to these comments and some of the editors’ comments, we have expanded on the policy implications specifically for cholera endemic vs. outbreak prone countries in the discussion paragraph four.

The missed burden of cholera is significant, as in all the settings where cholera is a public health problem, healthcare services are often accessed through informal routes. Without an estimate of these cases, asymptomatic or mild-to-moderately symptomatic cases and missed cases, a more holistic understanding of the true burden will not be forthcoming. The authors recommend that these should be accounted for using local contexts/data. Would it be possible for the authors to provide a little bit more framing around this, so that policy implementers in countries with novel and extended cholera outbreaks can try to identify a closer, locally valid estimate?

**Response**: Thank you for this comment. We have expanded on this in the discussion paragraph five. We are also glad to report that our team has received funding to carry out some of this work, which includes a systematic review of care seeking behavior for diarrhea and cholera (PROSPERO ID CRD42023402435).
