# Supplementary material for: Estimating the proportion of clinically suspected cholera cases that are true Vibrio cholerae infections: A systematic review and meta-analysis
Source: PLoS Med. 2023 Sep 14;20(9):e1004286. doi: 10.1371/journal.pmed.1004286 (PMC10538743; doi:10.1371/journal.pmed.1004286)
Supplement: Attachment — Submitted filename: Cholera_positivity_RTR_R2.docx [file pmed.1004286.s005.docx]

**Responses to Reviewers and Editors: Revision 2**

Requests from Editors:

GENERAL
Thank you for your detailed responses to previous editor and reviewer comments. Please see below for further comments that we require you address in full prior to publication.

COMPETING INTERESTS
All authors must declare their relevant competing interests per the PLOS policy, which can be seen here:
[https://nam02.safelinks.protection.outlook.com/?url=https%3A%2F%2Fjournals.plos.org%2Fplosmedicine%2Fs%2Fcompeting-interests&data=05%7C01%7Cazman%40jhu.edu%7C6da50b398d42437079f708db9b6e82ce%7C9fa4f438b1e6473b803f86f8aedf0dec%7C0%7C0%7C638274671167150110%7CUnknown%7CTWFpbGZsb3d8eyJWIjoiMC4wLjAwMDAiLCJQIjoiV2luMzIiLCJBTiI6Ik1haWwiLCJXVCI6Mn0%3D%7C3000%7C%7C%7C&sdata=Mpqa2p5PeungiVlp2OB9mgUQ3T17L0ruF2jZhOEPspc%3D&reserved=0](https://nam10.safelinks.protection.outlook.com/?url=https%3A%2F%2Fjournals.plos.org%2Fplosmedicine%2Fs%2Fcompeting-interests&data=05%7C01%7Ckirsten.wiens%40temple.edu%7C878eef5015714781397008db9b7d66ac%7C716e81efb52244738e3110bd02ccf6e5%7C0%7C0%7C638274735129962545%7CUnknown%7CTWFpbGZsb3d8eyJWIjoiMC4wLjAwMDAiLCJQIjoiV2luMzIiLCJBTiI6Ik1haWwiLCJXVCI6Mn0%3D%7C3000%7C%7C%7C&sdata=lfWgJPA6swXmOOJ57aG2tY32lZCJOKcDHw6XJ5hCGFE%3D&reserved=0)
For authors with ties to industry, please indicate whether any of the interests has a financial stake in the results of the current study.

**Response**: No authors declare competing interests. We have indicated this in the manuscript submission form.

We ask you to revise the title to better match journal style and suggest: "Estimating the proportion of Vibrio cholerae infections among suspected cholera cases: A systematic review and meta-analysis”.

**Response**: We have revised the title to “Estimating the proportion of clinically suspected cholera cases that are true *Vibrio cholerae* infections: A systematic review and meta-analysis”.

ABSTRACT
Please combine the methods and findings sections into one section, ‘Methods and findings’
Line 54 – ‘V. cholerae positivity was lower in studies with representative sampling and lower minimum ages in suspected case definitions.’ The use of the term lower (I think) in two different contexts is confusing, please revise for clarity.
Line 60 – please remove ‘and the resolution of the data’
Line 71 – please remove the funding statement and include only in the manuscript submission form.
In the main methods section (line 155) you state ‘…January 1, 2000 to reflect contemporary patterns in cholera positivity…’ please include the same or similar in the abstract to clearly justify your choice of search start date.

**Response**: We have made these revisions to the abstract.

AUTHOR SUMMARY
Thank you for including an author summary, which reads very nicely.
Line 91 – please place as the final bullet point of this sub-section.

**Response**: We inverted the order of the last two bullets in this sub-section.

INTRODUCTION
Line 112 - please define ‘PCR’ at first use for the reader.

**Response**: We have defined PCR.

METHODS and RESULTS
Line 202 – please move this statement to the beginning of the methods section.
Currently, the ethics approval appears to be quoted both at the start and end of the Methods section: please de-duplicate.

**Response**: We moved the PRISMA statement to the beginning of the Systematic Review Methods sub-section. We also removed the ethics statement at the end of the Methods.

TABLES
Please include a table which summaries the basic information of the studies utilized for your review – author, year of publication, country, study type/design, number of ppts, diagnostic test as a minimum.

**Response**: We decided to not include a table in the supplement summarizing each individual study due to the large number of studies included (n = 119). This table would cover many pages and would likely not be informative. Instead, we have included all studies including citation, dates, country, study type/design, number of ppts, diagnostic test type, among many other variables in an Excel sheet in S1 Data. Including this information in an Excel sheet allows for filtering by study type, method, etc., and ensures reproducibility of our results. This data is also available in csv format in the public GitHub repository: <https://github.com/HopkinsIDD/cholera_positivity/tree/main/data/extracted_data>

Please include an assessment of study quality.

**Response**: Existing frameworks such as the Cochran risk-of-bias assessment tools (i.e., RoB 2 and ROBINS-I) for assessing study quality are focused on risk of bias in clinical trials and observational studies aimed at assessing the effect of an exposure (e.g., vaccine or biologic) on a specific disease outcome. Given that the primary aim of our study is proportion positive, which is almost never a primary endpoint in published studies, these developed scales are not well adapted. Instead we focused our assessments on data quality checks and analyses to assess the impact of study methodology on our outcome (i.e., *V. cholerae* positivity), particularly sampling method quality, detailed below.

Our methods for data quality checks are detailed in the Methods on lines 205-209. In addition, we assessed the potential for different methods to bias our results, detailed in the Methods on lines 210-218, corresponding to results in Figure 2 and Figure S3. Finally, we assessed and grouped studies by quality of sampling method (i.e., whether they used population-representative sampling). We examined the impact of sampling quality on our results, which is detailed in lines 318-321 (Figure 2B), 352-360 (Figure 3; Table S4), and 368-375 (Table S5). Adjusted and reported *V. cholerae* positivity for each study, with details of each study’s sampling quality, are also shown in Figure 4.

Table 1 – please provide an appropriate caption which clearly details the table content without the need to refer to the text. What do you mean by ‘Number of observations’ the ‘observations’ confuses me. Could it simply read number?

**Response**: To be clear that we are not referring to the number of studies, but the number of unique rows of data (or ‘observations’) that went into the model, we feel it’s important for clarity to include ‘observations’ in the caption, along with a definition, which we have added. We have simplified the table itself to just read ‘number’ and ‘percent’.

FIGURES
In the captions for example line 922 you refer to ‘observations’ here also. This term is not used in the text. Do you mean observed cases? Please clarify/amend and in consistency with table 1.

**Response**: Our definition of ‘observations’ is detailed on lines 283-286. We have added this definition to the figure and table legends.

Figures 2, 3 and 4 please define all abbreviations in either a footnote or in the figure caption – PCR, RDT, IQR, V.cholera

**Response**: We have defined all abbreviations in Figures 2-4.

Figure 3 – please detail the meaning of the dots and lines in the figure caption.

**Response**: We have edited the legend to clarify this.

DISCUSSION
Lines 48 and 443 – please remove these statements from the end of the discussion and include only in the manuscript submission form they will be compiled as metadata.

**Response**: We have removed the funding and competing interests statements from the manuscript and included them in the manuscript submission form.

SUPPORTING INFORMATION
Please cite, label and upload your Supporting Information as outlined here: [https://nam02.safelinks.protection.outlook.com/?url=https%3A%2F%2Fjournals.plos.org%2Fplosmedicine%2Fs%2Fsupporting-information&data=05%7C01%7Cazman%40jhu.edu%7C6da50b398d42437079f708db9b6e82ce%7C9fa4f438b1e6473b803f86f8aedf0dec%7C0%7C0%7C638274671167150110%7CUnknown%7CTWFpbGZsb3d8eyJWIjoiMC4wLjAwMDAiLCJQIjoiV2luMzIiLCJBTiI6Ik1haWwiLCJXVCI6Mn0%3D%7C3000%7C%7C%7C&sdata=AIQQcz0D80Xb1BDdfJUWNL2n0ygPWfsXT4vJHPkN%2FyI%3D&reserved=0](https://nam10.safelinks.protection.outlook.com/?url=https%3A%2F%2Fjournals.plos.org%2Fplosmedicine%2Fs%2Fsupporting-information&data=05%7C01%7Ckirsten.wiens%40temple.edu%7C878eef5015714781397008db9b7d66ac%7C716e81efb52244738e3110bd02ccf6e5%7C0%7C0%7C638274735129962545%7CUnknown%7CTWFpbGZsb3d8eyJWIjoiMC4wLjAwMDAiLCJQIjoiV2luMzIiLCJBTiI6Ik1haWwiLCJXVCI6Mn0%3D%7C3000%7C%7C%7C&sdata=LIhz%2BpKySZCQ2wz1IxhVAu6M1RtU06HGbj7%2FNOU8vmw%3D&reserved=0)

**Response**: We have updated the citations and labels and added legends for the supporting information.

Please ensure that the reference format follows that of our guidance which can be found here. [https://nam02.safelinks.protection.outlook.com/?url=https%3A%2F%2Fjournals.plos.org%2Fplosmedicine%2Fs%2Fsubmission-guidelines%23loc-references&data=05%7C01%7Cazman%40jhu.edu%7C6da50b398d42437079f708db9b6e82ce%7C9fa4f438b1e6473b803f86f8aedf0dec%7C0%7C0%7C638274671167150110%7CUnknown%7CTWFpbGZsb3d8eyJWIjoiMC4wLjAwMDAiLCJQIjoiV2luMzIiLCJBTiI6Ik1haWwiLCJXVCI6Mn0%3D%7C3000%7C%7C%7C&sdata=mThoIsY3Wpz3DnYFAJbQp8J6BHzmMYoXxgayprf30I4%3D&reserved=0](https://nam10.safelinks.protection.outlook.com/?url=https%3A%2F%2Fjournals.plos.org%2Fplosmedicine%2Fs%2Fsubmission-guidelines%23loc-references&data=05%7C01%7Ckirsten.wiens%40temple.edu%7C878eef5015714781397008db9b7d66ac%7C716e81efb52244738e3110bd02ccf6e5%7C0%7C0%7C638274735129962545%7CUnknown%7CTWFpbGZsb3d8eyJWIjoiMC4wLjAwMDAiLCJQIjoiV2luMzIiLCJBTiI6Ik1haWwiLCJXVCI6Mn0%3D%7C3000%7C%7C%7C&sdata=AQhBfcbZugMpQUqJoX3kGwDACoSkz6esL4eCvN%2Bw4Ho%3D&reserved=0)

**Response**: We have updated the reference in the Supporting Information to match PLoS Medicine style.

Figures – please define abbreviations RDT and PCR throughout in the caption or in a footnote.

**Response**: We have defined these throughout.

Please use the form "PLoS" in the reference list; and revisit reference 88, which appears to be missing publication information.

**Response**: We have edited PLOS to PLoS and added publication information to reference 88.

SOCIAL MEDIA
To help us extend the reach of your research, please detail any Twitter handles you wish to be included when we tweet this paper (including your own, your coauthors’, your institution, funder, or lab) in the manuscript submission form when you re-submit the manuscript.

**Response**: You can use: @KirstenWiens @andrewazman [@JHIDDynamics](https://twitter.com/JHIDDynamics) [@JohnsHopkinsSPH](https://twitter.com/JohnsHopkinsSPH) [@TempleEpiBio](https://twitter.com/TempleEpiBio) [@TempleCPH](https://twitter.com/TempleCPH)

Comments from Reviewers:

*** Reviewer #1:

Thanks for the revised manuscript and replies to my original review.

With the addition of the new data, some of the STAN models needed modification. This sounds like a reasonable strategy to deal with the heterogeneity.

The manuscript looks good from my perspective, the figures are great (colour scheme looks fine to me).

**Response**: We thank the reviewer for these comments.

***

Any attachments provided with reviews can be seen via the following link:

**Response**: We did not see any additional reviewer comments attached.
